# Uterine Hydatids

**Published:** 1849-03

**Authors:** 


### OBSTETRICS.

*Uterine Hydatids.*—At the Pathological Society of London, Dr. Ramsbotham exhibited a specimen of the, so-called, Uterine Hydatids, about a pint in quantity, that had been expelled under his superintendence the previous evening. The patient, 28 years old, had been married twice: to her first husband only ten months; to her second two years; and had never either borne a child or aborted. The catamenial discharge was perfectly regular till the July period, when twelve days beyond the usual time passed without any appearance showing itself. There then came on, however, a slight sanguineous weeping from the uterus, which continued almost without intermission, though to a very trifling extent. As the abdomen was evidently getting larger, Dr. R. was consulted, on October 27th, to determine whether she was pregnant. He found the discharge still going on, and the uterus as large as one between four and five months gravid. On examination per vaginam the cervix was felt to be considerably developed, and the os uteri gave to the finger the characteristics of pregnancy. His opinion was that the uterus contained something, probably an ovum, whether living or dead could not be ascertained, and that probably she would abort within a short time; she was directed to keep her bed. He heard nothing more of her till last night, the 19th, when he was summoned hastily, and found the mass expelled: she had had periodical uterine pains for three or four hours, and had suffered some hæmorrhage, but not enough to effect the system sensibly. The uterus had subsided almost entirely within the pelvic cavity, and was quite empty. The bleeding had nearly ceased, and she was in no pain.

Dr. R. remarked that it was a doubt still with some physiologists whether these bodies were true hydatids or not; he considered they were not so, because the cysts contained nothing like the *echinococci.* He looked upon them as being merely a diseased, dilated, or preternaturally developed, condition of the villi of the chorion; because, under the microscope, their terminal vessels are seen to have exactly the same arrangement as the vessels of those villi possess, and which are delineated faithfully by Wagner and Weber. He never saw them expelled from a virgin uterus, though many cases had come under his notice, and believed them the result of impregnation. This fact, however, would certainly appear to be rendered somewhat doubtful by two cases recorded, fourteen or fifteen years ago, by Dr. Andrew, in the Glasgow Medical Journal, where some were voided by unmarried girls of good character; in one of which subjects there was present, according to Dr. Andrew, a perfect hymen.

Dr. Ramsbotham also presented an organized mass, of the size of a small orange, which was attached to the umbilical cord of a fine-grown infant (born about a month ago,) close to its origin from the placenta, by a pedtcle of about half an inch in length. It was covered by perfectly formed cutis and cuticle, was made up of cellular substance with fat, and of gelatinous matter contained in small cells, and at one part near the surface there was a quantity of half formed bones; it floated in the liquor amnii surrounding the live child, was fed by its vessels, and was evidently an abortive attempt at the formation of a second fœtus.—*Ibid.*

